# XANES reference library of sulphur-containing compounds for biological research: a status report from the ASTRA beamline at the SOLARIS National Synchrotron Radiation Centre

**DOI:** 10.1039/d5ra00682a

**Published:** 2025-04-28

**Authors:** Agnieszka Klonecka, Joanna Sławek, Grzegorz Gazdowicz, Alexey Maximenko, Andrzej Sławek, Marcel Piszak, Henning Lichtenberg, Maciej Kozak

**Affiliations:** a SOLARIS National Synchrotron Radiation Centre Kraków Poland Joanna.slawek@uj.edu.pl; b The Faculty of Physics, Astronomy and Applied Computer Science, Jagiellonian University Kraków Poland; c Doctoral School of Exact and Natural Science, Jagiellonian University Kraków Poland; d AGH University of Science and Technology, Academic Centre for Materials and Nanotechnology Kraków Poland; e Hochschule Niederrhein University of Applied Sciences Krefeld Germany; f Department of Biomedical Physics, Faculty of Physics, Adam Mickiewicz University Poznań Poland

## Abstract

Sulphur is present in a vast multitude of biological compounds, and X-ray absorption spectroscopy (XAS) is a powerful and well-established characterization technique to study the local atomic environment of this chemical element in such systems in detail, with a high potential for advancing knowledge in medicine, biotechnology and environmental research. For this project, X-ray absorption near edge structure (XANES) spectroscopy at the sulphur K-absorption edge was used to investigate sulphur-containing compounds of biological interest at the ASTRA beamline (SOLARIS National Synchrotron Radiation Centre, Kraków, Poland). XANES provides valuable insights into the bonding environment of sulphur and thereby contributes to a deeper understanding of the structural characteristics and functional roles of sulphur in biological systems. The ASTRA beamline, specifically optimized for XAS in the tender energy range, offers ideal conditions for further research in this field, enabling detailed analysis of the composition and changes in sulphur containing compounds.

## Abstract 2

XANES spectra of sulphur compounds measured at ASTRA beamline (SOLARIS) support a growing reference library for biological research, enabling detailed insights into sulphur’s roles in medicine, biotech, and environmental studies.

## Graphical abstract

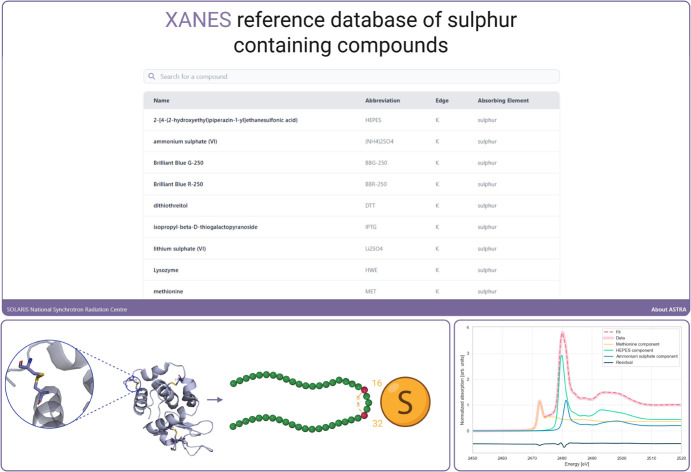

## Introduction

X-ray Absorption Spectroscopy (XAS) and its sub-techniques – Extended X-ray Absorption Fine Structure (EXAFS) and X-ray Absorption Near Edge Structure (XANES) – has been widely used for many years in the study of myriads of inorganic systems, such as catalysts, numerous metal-containing complexes, minerals, and composite materials, as well as various biological systems.^1–4^ In recent years, biological XAS studies, combined with *ab initio* calculations, have increasingly focused on the determination of the local structure of binding sites in protein complexes with metal ions,^5–7^ the observation of structural changes at metal sites during catalytic processes,^8^ and the structure of inorganic clusters involved in catalytic processes in numerous metalloenzymes or interactions with ligands.^9^

Studies on the accumulation of metal ions in tissues, their local structure and their role in the development of certain diseases, including neurodegenerative pathology (*e.g.*, Alzheimer's disease,^10–12^ Creutzfeldt–Jakob disease,^13,14^ Parkinson's disease^15^), are also of great interest.

It is noteworthy, however, that XAS measurements of organometallic and bio-metallic systems at high photon energies are currently widely and easily available at most synchrotron facilities in Europe and worldwide, whereas XAS studies at medium and low energies (1–5 keV) represent a niche that can be filled by the experimental systems offered at low-energy synchrotrons. It is worth mentioning that an important application of XAS is the study of the local atomic environment of sulphur in biological systems by recording absorption spectra at the sulphur K-edge (2.472 keV). Such measurements complement, among other methods, structural characterization of sulphur-containing systems using infrared^16^ and Raman spectroscopy.^17^

Sulphur atoms are present at the active centres of some enzymes (*e.g.* cysteine proteases) and at metal binding sites (*e.g.* zinc). The study of the spatial structure of these systems (focusing on the vicinity of S-atoms or disulfide bonds) using XAS spectroscopy^18^ represents an interesting alternative to FTIR studies, as it provides insight into the environment of sulphur atoms. Compared to FTIR, XAS does not require elaborate sample preparation, such as adjusting the sample thickness since it has the ability to probe deeper into the sample and can often be performed under conditions closer to the natural physiological environment.

In addition, in FTIR the absorption bands are low, therefore XANES provides more complex information of biologically relevant sulphur compounds (*e.g.* methionine, cysteine/glutathione, cysteine/oxidized glutathione) are inherently weak due to the smaller dipole moment of thio-ether, thiol and disulfide functional groups. In addition, these bonds often overlap with absorption bands of other dominant functional groups in biological samples (*e.g.* carbohydrates or phosphates).^18,19^ Therefore, the elemental specificity of XANES spectra is a major advantage when studying sulphur in complex samples.^20,21^

It is worthwhile to take advantage of the experimental capabilities available at medium-energy synchrotron facilities, such as the ASTRA beamline at the SOLARIS National Synchrotron Radiation Centre.^22^ The ASTRA beamline was specifically designed for XAS measurements in the tender photon energy range, down to 1 keV (Na K-edge).^23^ In combination with the spectral flux distribution of the bending magnet at the 1.5 GeV SOLARIS storage ring (characteristic energy ∼2 keV) this beamline is specifically attractive for this range, including the sulphur K absorption edge. To minimize absorption of low energy photons the beamline is windowless down to the exit flange of the monochromator, where just one thin foil window separates the monochromator's high vacuum from the ionization and sample chambers. Direct access to synchrotron beamlines optimized for the energy range close to the sulphur K-edge is also important for interdisciplinary research. However, an important aspect of these studies, in addition to the availability of the appropriate infrastructure, is a standardized, reproducible methodology and suitable XAS reference data. It is expected that combining XAS with Raman spectroscopy as complementary technique will provide highly valuable results, especially since a recently installed Raman micro spectrometer is now under commissioning at the ASTRA beamline. Since assuming full user operation in late 2023, ASTRA has become highly appreciated by the users. The large number of high quality XAS spectra at different absorption edges recorded since then calls for a comprehensive library of reference spectra, which is under development now. In the future the XANES library will be supplemented with corresponding Raman spectra.

Therefore, the aim of this project was to create a library of sulphur K-edge XAS spectra of substances widely used in molecular biology, biochemistry or biophysics, including buffers, dyes and reducing agents. As a starting point, we collected a set of 13 X-ray absorption spectra and analysed them in detail. Here, we also present the application of *in silico* calculations providing deeper understanding of the resulting spectra and demonstrate the potential of linear combination analysis using our library as a source of reference spectra.

Practical considerations for performing XAS experiments on biological systems have been discussed previously.^24^ In past years, research at synchrotrons around the world has provided valuable insights into metal homeostasis, redox regulation, and protein interactions. At the Stanford Synchrotron Radiation Lightsource (SSRL), studies at beamline 4–3 have focused on the development of a sulphur XANES model library with applications in brain tissue analysis.^25^ Interdisciplinary research combining XANES and FTIR at beamline 14–3 at SSRL has allowed comparisons between disulfide levels (XANES) and protein aggregates (FTIR).^26^ The Soft X-ray Microcharacterization Beamline (SXRMB) at the Canadian Light Source (CLS) has contributed to sulphur XANES studies, expanding our understanding of sulphur speciation in biological samples.^27^ In addition, research at the Australian Synchrotron's XAS beamline has characterized thiol and disulfide levels in brain tissue under oxidative and non-oxidative conditions.^28^ At the Center for Advanced Ultrastructural Research at the University of Georgia, studies have explored the sulphur bonding environment in Fe–S clusters of proteins, particularly in relation to coordination chemistry.^29^ Similarly, at the European Synchrotron Radiation Facility (ESRF), studies at the ID21 beamline have provided insight into sulphur coordination in Fe–S protein clusters.^28^

Taken together, these studies underscore the importance of synchrotron-based XAS techniques in advancing our understanding of complex biochemical mechanisms in health and disease. Systematic approaches to the development and use of reference libraries, such as those presented here, are critical to improving the interpretation of sulphur XANES spectra in biological and inorganic chemistry.

The potential for interdisciplinary studies using XAS and its combinations with complementary methods has been emphasised in recent works.^30–33^ This offers further opportunities to gain detailed molecular insights, particularly for sulphur-containing systems, where alternative techniques often fall short. The availability of medium energy experimental stations, such as the ASTRA beamline at the SOLARIS National Synchrotron Radiation Centre, is expanding access to sulphur K-edge studies allowing structural characterisation under physiologically relevant conditions.

## Methodology

### Selection of compounds for S-XANES library

The main criterion for the selection of reference compounds for this study was their use in routine research in the field of biochemistry, molecular biology, biophysics or protein crystallography. Thirteen different biological compounds containing sulphur in different chemical states were initially selected. Two substances (Coomassie® Brilliant Blue R-250 and Coomassie® Brilliant Blue G-250) were chosen to represent dyes for biological research. They are commonly used to detect proteins after electrophoresis.^34,35^

Sodium dodecyl sulphate (SDS) is an anionic surfactant used in molecular biology for cell membrane disruption, nucleic acid hybridisation, DNA extraction and protein electrophoresis.^36–38^ Dithiothreitol (DTT) is used as a component of biological buffers in protein purification and analysis.^36,39^ Isopropyl-β-d-thiogalactopyranoside (IPTG), is an inducer of β-galactosidase activity in *Escherichia coli* and routinely used in protein expression.^40^

Another group of compounds commonly used in molecular biology are Good's buffers.^41^ Representatives of this group are the sulphur-containing compounds *N*-cyclohexyl-2-aminoethanesulphonic acid (CHES) and 4-(2-hydroxyethyl)-1-piperazineethanesulphonic acid (HEPES).^42^

Sulphur-containing salts are widely used in molecular and structural biology. Ammonium sulphate (VI) is widely used in protein crystallography and other industrial applications.^43,44^ Nickel sulphate (VI) and lithium sulphate (VI) are additives in many buffers and often used, for example, in structural studies of proteins, increasing enzymatic stability and altering the morphology of protein crystals.^45^

Finally, hen egg white lysozyme was chosen as a model example of a sulphur containing protein.^46^

### Materials

The tested compounds purchased from commercial sources are listed in Table 1. Information about suppliers and availability as well as their normalized XAS spectra along with their first and second derivatives are included in the library at the ASTRA beamline or can be requested from the corresponding author.

**Table 1:** Tested compounds and their suppliers

Number	Compound name	Short name
1	Isopropyl-β-d-thiogalactopyranoside	IPTG
2	Brilliant Blue R-250	BBR-250
3	*N*-Cyclohexyl-2-aminoethanesulphonic acid	CHES
4	Dithiothreitol	DTT
5	*N*-(2-Hydroxyethyl)piperazine-*N*′–(2-ethane sulphonic acid)	HEPES
6	Brilliant Blue G-250	BBG-250
7	Ammonium sulphate (VI)	(NH_4_)_2_SO_4_
8	Sodium dodecyl sulphate	SDS
9	Lithium sulphate (VI)	Li_2_SO_4_
10	Nickel sulphate (VI)	NiSO4_4_
11	Methionine	MET
12	Zinc sulphate (VI)	ZnSO_4_
13	Hen egg white lysozyme	HEW

### Sample preparation

XAS spectra of all samples except lysozyme were measured in transmission mode. Since they were available in powder form and therefore could be prepared as sufficiently thin, homogeneous layers without a heavy element matrix. One of the main criteria for selecting either transmission or fluorescence mode for XAS measurements is the absorber concentration. For concentrations of above *ca.* 5 weight%, transmission mode is preferable as it provides XAS spectra free of self-absorption artefacts with high signal to noise ratio.

Accurate XAS measurements in transmission mode require uniformity of the sample thickness across the beam spot.^47^ Therefore, each reference compound was ground to a fine powder using an agate mortar and pestle. A commonly used method for XAS sample preparation involves pressing optimised amounts of sample material and a suitable binder to pellets. The optimum amount of material corresponds to approximately one absorption length of the element of interest through the sample. This gives the best signal level and signal to noise ratio. The purpose of the binder is to provide rigidity and integrity to the pellet while having negligible effects on the spectra obtained.

Performing XAS measurements in transmission mode in the tender energy range requires a specific approach to sample preparation, for two reasons: firstly, the minimum amount of binder required to obtain a usable, handy pellet should be considered. By way of an over-estimate, it could be said that this amount would result in a pellet of less than 0.1 mm thickness. Even at this thickness, commonly used binders such as cellulose, starch or boron nitride significantly absorb X-rays around the S K-edge.^48^ The second problem is the extremely small amount of sample material corresponding to one absorption length along the beam – the optimum amount of material for the sulphur K-edge has been established to be a few milligrams. A commonly used solution to overcome these problems is to spread the sample powder on a thin self-adhesive tape.^47^ This method has the advantage of low substrate absorption and the possibility to prepare a very thin, uniform layer of sample material.

For this project, 1 mil (26 μm) thick Kapton tape was used. Including the thickness of the glue layer, according to the manufacturer, the total thickness of the substrate is 63 μm. The samples used for this study, ground to fine powder in the mortar, were pressed firmly onto the Kapton tape using a steel spatula to improve adhesion and homogeneity of the sample. Excess material was removed using cotton buds and a soft brush. To avoid artefacts in the spectra from supporting materials, reference spectra of blank Kapton tape (without sample) were recorded prior to the experiment and its purity was checked.

An obvious disadvantage of this sample preparation technique is the lack of precise control over the amount of sample. Since the amount of powder to be attached to the tape has an upper limit, to achieve optimal sample thickness it was sometimes necessary to stack several layers of Kapton covered with the sample material. The optimum number of layers in the stack was determined empirically by aiming for an edge step in the resulting XAS spectra as close to 1 as possible.

Lysozyme as a compound with lower sulphur concentration had to be measured in fluorescence mode to obtain a sufficiently high absorption step. In biological research it is crucial to study molecules in their native form. To approach the conditions, present in living organism, lysozyme was measured in aqueous solution. Powdered hen egg white lysozyme was dissolved in distilled water at a concentration of 100 mg ml^−1^. Approximately 5 μl of solution was applied between two pieces of 3 μm polypropylene foil and placed in a specially designed liquid sample holder.

For providing an example of linear combination analysis (LCA), a sample consisting of three sulphur-containing compounds was prepared: ammonium sulphate, HEPES and methionine – substances commonly used in life science research. The substances were separately ground in an agate mortar for 5 minutes, then mixed and ground together for another 5 minutes in ambient atmosphere. The final powder was spread on adhesive tape. Excess material was removed with cotton buds.

### X-ray absorption experimental study

The XANES spectra were recorded at the ASTRA beamline at the SOLARIS synchrotron (Kraków, Poland). ASTRA is using synchrotron radiation from a bending magnet (1.31 T) and a double crystal monochromator equipped with InSb (111) crystals for XAS measurements at the sulphur K-edge. The beam has a rectangular shape – 1 mm high and 10 mm wide. During all measurements performed in transmission mode X-ray intensities were measured using ionisation chambers filled with nitrogen gas at a pressure of about 25 torr. The monochromator was calibrated using a ZnSO_4_ reference sample with a well-defined white line peak at 2481.4 eV in the absorption spectrum. The energy calibration of the beamline was frequently checked between XAS scans by replacing the actual sample with a reference sample (ZnSO_4_) to check for shifts in energy calibration. Corresponding alignment was applied if needed. No energy shifts were observed between individual scans of the same sample.

Fluorescence mode XANES spectra were collected using a one element Ketek H150 SDD detector with AXAS-M1 preamplifier and AXAS-M2 signal processing unit. The detector was oriented at 90° to the incoming beam, and the sample at 45°. All experiments were performed at room temperature using the experiment control software AstraLibra, a LabVIEW-based program developed at SOLARIS specifically for the ASTRA beamline.^49^

**Fig. 1:**
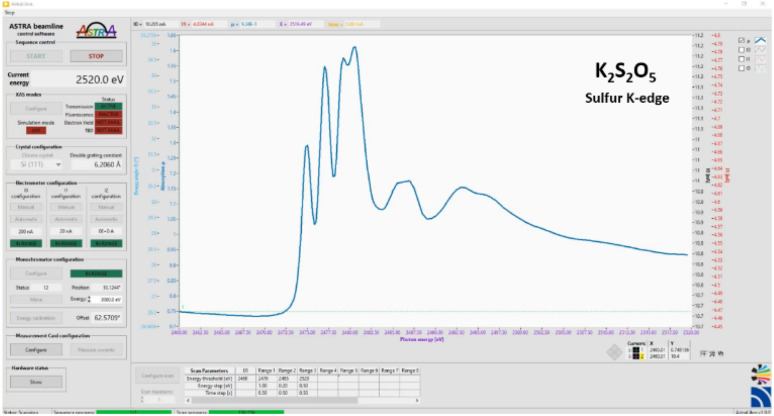
Front panel of the AstraLibra experiment control software.

It was originally based on a software package provided by the Synchrotron Light Research Institute (SLRI, Thailand). The software controls the Bragg axis rotation of the double crystal monochromator for energy scanning during XAS measurements. To achieve best performance of scanning accuracy, AstraLibra supports custom PID and motion control parameters setup. The position error threshold was set to 0.0005°. The ionization chambers' currents are converted to analogue voltage signals by Keithley 6514 electrometers and then sampled by a Measurement Computing USB-1604HS DAQ card at a rate of 5 kHz. Data acquisition is continuous, and integration is triggered by the Bragg axis motion controller when the desired Bragg angle, *i.e.* the target photon energy, is reached. Each hardware component controlled by AstraLibra is represented by an independent module, controlling device initialization, configuration, and communication. Data is gathered and visualized on the main control panel (Fig. 1) and simultaneously saved to a custom file format compatible with the Athena software for XAS analysis. AstraLibra is based on Queued Message Handler architecture allowing for parallel execution of various tasks.^49^ The scanning parameters for the monochromator were chosen as follows: 0.5 eV steps in the pre-edge region (2440–2468 eV), 0.2 eV in the near edge region (2468–2485 eV), 0.3 eV steps in the post-edge region (2485–2520 eV) and 1 eV steps in the energy range from 2520 to 2570 eV. Integration time was 0.75 s in all intervals. The samples were measured three times, each scan took around 8 minutes. The resulting spectra were averaged. No significant differences between single spectra of the same compound were observed. Thus, no signs of chemical changes or damage in the samples due to X-ray irradiation was detected. Radiation damage is unlikely to occur at the ASTRA beamline due to the relatively large macroscopic beam cross section (unfocused beam, 1 × 10 mm) resulting in a low photon density and relatively large, irradiated sample surface. Nevertheless, a series of measurements was conducted to check the sample stability in the case of lysozyme. The atmosphere of pure (5N) nitrogen in ASTRA's sample chamber additionally contributes to maintaining chemical stability during measurements. Background subtraction and normalization of raw XAS data as well as data analysis were performed using the Athena software.^50^ Since XANES spectra are additive, the spectrum of a sample containing a mixture of different specifications of the element of interest can be fit to a linear combination of the spectra of the individual components, or spectra of reference compounds with similar speciation. The coefficients in this linear combination correspond to the molar ratios of these species (relative concentrations). Linear combination fitting was performed with the restriction that the weights of each component range between zero and one. It was not required that the weights sum to 100% to allow some freedom to compensate for minimal differences in the normalisation of the components. Nevertheless, the sum of the components' contributions was 99.8% after the fitting.

### X-ray absorption simulation study

Calculated XANES spectra were obtained from the Finite Difference Method Near Edge Structure (FDMNES) software^51–53^^.^ This simulation code uses Density Functional Theory (DFT) with Local Spin Density Approximation (LSDA). Positions of the atoms were taken from atomic coordination files (cif-files) from the Cambridge Crystallographic Data Centre (CCDC) database.^54,55^ Each single molecule was excised from the structure and inserted in a 20 × 20 × 20 Å simulation box. Sulphur K-edges were calculated with the Finite Difference Method for XAFS (FDMX)^53^ using dipole electronic transitions, where the orbital quantum number may change by 1 (Δ*l* = ±1). The cluster radius was set to 6 Å. Spin–orbit coupling effects and relativistic effects were omitted. The presented spectra were Lorentzian convoluted. Broadening width increases with energy as an arctangent. The default parameters were used: 0.59 eV for hole, 15 eV for max, 30 eV for Ecent, and 30 eV for Elarg.

## Results and discussion

By using XANES it is possible to retrieve information about the bonding environment of sulphur. To establish a comprehensive library of reference X-ray absorption spectra for sulphur-containing compounds frequently employed in biological research, sulphur K-edge XANES spectra of suitable reference compounds were collected.

The substances included in the library represent diverse chemical states and coordination environments of sulphur. Among these compounds were dyes, buffers, salts, and other common substances, which can affect the purity and structure of samples used for XAS studies of biological systems.

**Fig. 2:**
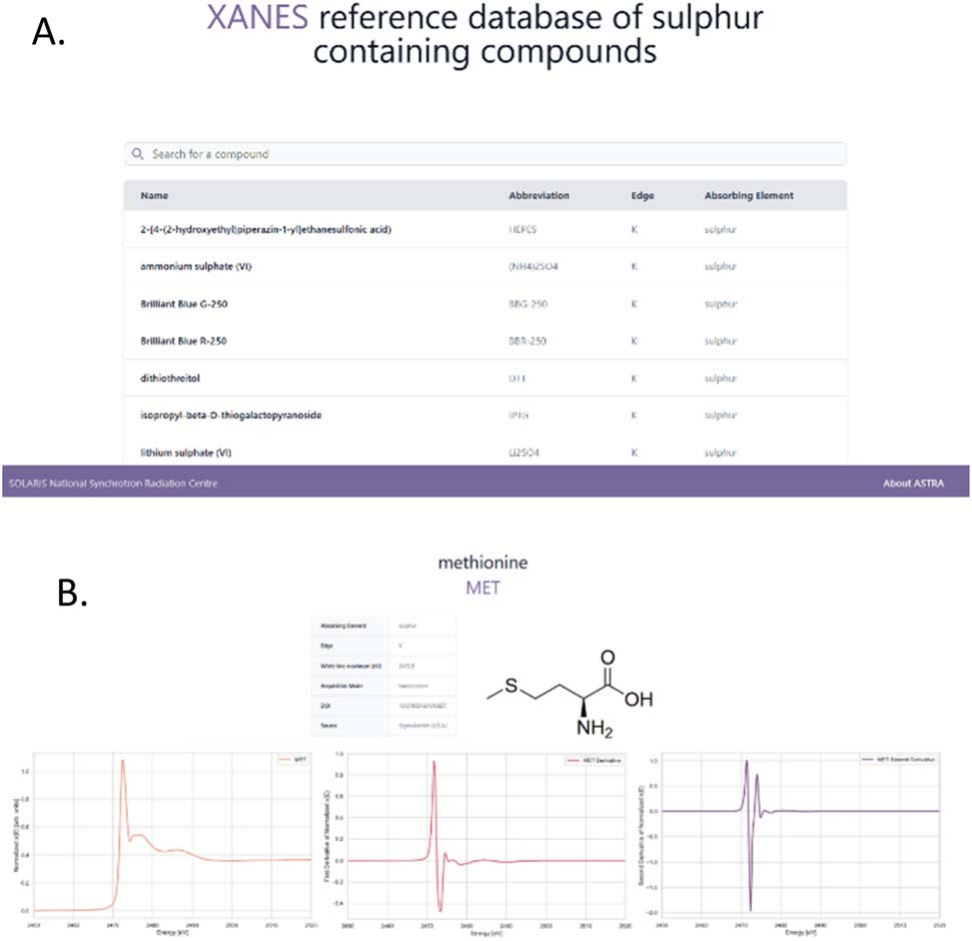
XANES reference database of sulphur containing compounds; (A) homepage, (B) methionine compound page at ASTRA website.

The resulting spectra, along with the data acquisition mode, chemical element of interest, white line maximum positions [eV], and the first and second derivatives, have been compiled into a database currently accessible to SOLARIS NSRC users (Fig. 2). In the future, we aim to expand access and make the extended database available to a broader community.

### Library of biological compounds preparation

Table 2 shows the sulphur environment in all tested compounds along with the energy positions of the white line maxima in the measured XANES spectra.

**Table 2:** Sulphur environments and values of experimental white line peak positions of tested compounds. The first and second derivatives of each compound's XANES spectrum are included in the created library. The first derivative was used to determine the energy position of the white line maximum

Compound	Sulphur environment	Position of white line maximum [eV]
IPTG	R–S–R	2472.5
DTT	R–SH	2472.6
MET	R–S–R	2472.9
CHES	R–S(O)_2_OH	2480.0
HEPES	R–S(O)_2_OH	2480.0
BBG-250	R–S(O)_2_OH	2480.6
BBR-250	R–S(O)_2_OH	2480.6
NiSO_4_	SO_4_^2−^	2481.3
(NH_4_)_2_SO_4_	SO_4_^2−^	2481.5
Li_2_SO_4_	SO_4_^2−^	2481.9
SDS	SO_4_^2−^	2482.0

Sulphur K-edge XANES spectra are strongly influenced by the oxidation state (valency) of sulphur, but also by further details of its bonding environment. Spectral features, including the position of the white line, can vary even between samples with sulphur in very similar coordination geometry.^56^

**Fig. 3:**
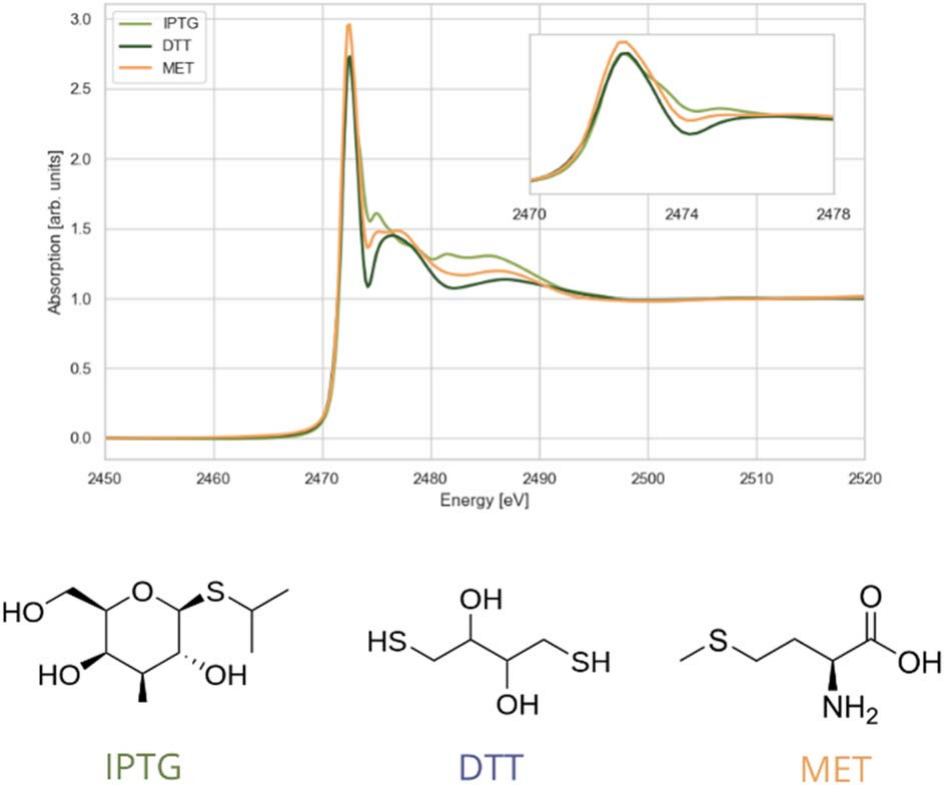
Sulphur K-edge XANES spectra of compounds with sulphur environment R–S–R; IPTG (isopropyl-β-d-thiogalactopyranoside), DTT (dithiothreitol), MET (methionine). The inset displays the white lines at a larger scale.

The first group of compounds analysed contain sulphur in an R–S–R environment (where R is a functional group). IPTG, DTT, and MET were selected as representative examples (Fig. 3) due to their varying distances between sulphur and nearby oxygen atoms. The energy positions of the white line maxima in the XANES spectra of these compounds fall within the range between 2472.5 and 2472.9 eV. While the overall spectral shape is similar across the compounds, notable differences are observed between 2473.0 and 2482.5 eV. These variations are influenced by the immediate chemical environment of the sulphur atom. Chemically, IPTG is an *S*-glycosyl compound with an isopropyl group attached to the anomeric sulphur, whereas MET features sulphur bonded to two alkyl groups. In DTT, sulphur is surrounded by one alkyl group and one thiol group. The position of the white line is sensitive to the local electronic and structural environment of the absorbing atom, in this case sulphur. A higher effective nuclear charge of the sulphur atom affects the binding energy of core electrons, causing a shift of the white line maximum towards higher energy values. This tendency is pronounced in the spectra of the investigated compounds: in IPTG the white line maximum occurs at 2472.5 eV, followed by DTT (2472.6 eV), and finally MET (2472.9 eV).

**Fig. 4:**
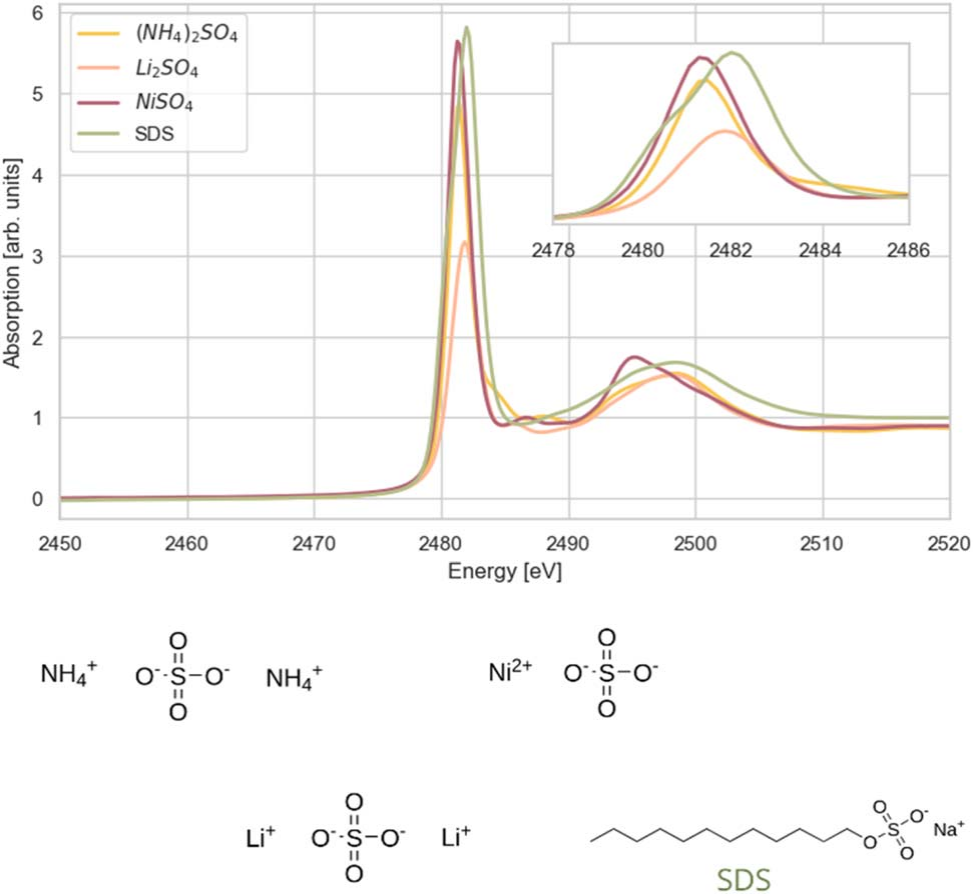
Sulphur K-edge XANES spectra of compounds with sulphur environment R–S(O)_2_OH; CHES (*N*-cyclohexyl-2-aminoethanesulphonic acid), HEPES (*N*-(2-hydroxyethyl)piperazine-*N*′-(2-ethane sulphonic acid)), BBR-250 (Brilliant Blue R 250), BBG-250 (Brilliant Blue G 250). The inset displays the white lines at a larger scale.

The second group of samples represents R–S(O)_2_OH environment. CHES HEPES, BBG-250, and BBR-250 were selected for analysis, as they all contain a sulfonic acid group but differ in the second group attached to the sulphur atom (Fig. 4).

In CHES and HEPES, the sulphur atom is bonded to an alkyl group that connects to a nitrogen atom. For this bond the position of the white line maximum is at 2480.0 eV.

However, in HEPES, the nitrogen atom is part of a ring structure, whereas in CHES, the nitrogen is attached to a cyclohexane group. These structural differences influence the post-edge spectral features in the 2482.0–2490.0 eV region.

The other two compounds, BBG-250 and BBR-250, exhibit only slight structural variations, located several coordination spheres away from the absorber atom. As expected, these differences do not significantly affect the main spectral features, including the position and intensity of the white line and the weaker local absorption maximum at 2487.5 eV. However, they appear to slightly influence the broader feature observed at higher energies, centred around 2497.4 eV, corresponding to multiple scattering resonances.

**Fig. 5:**
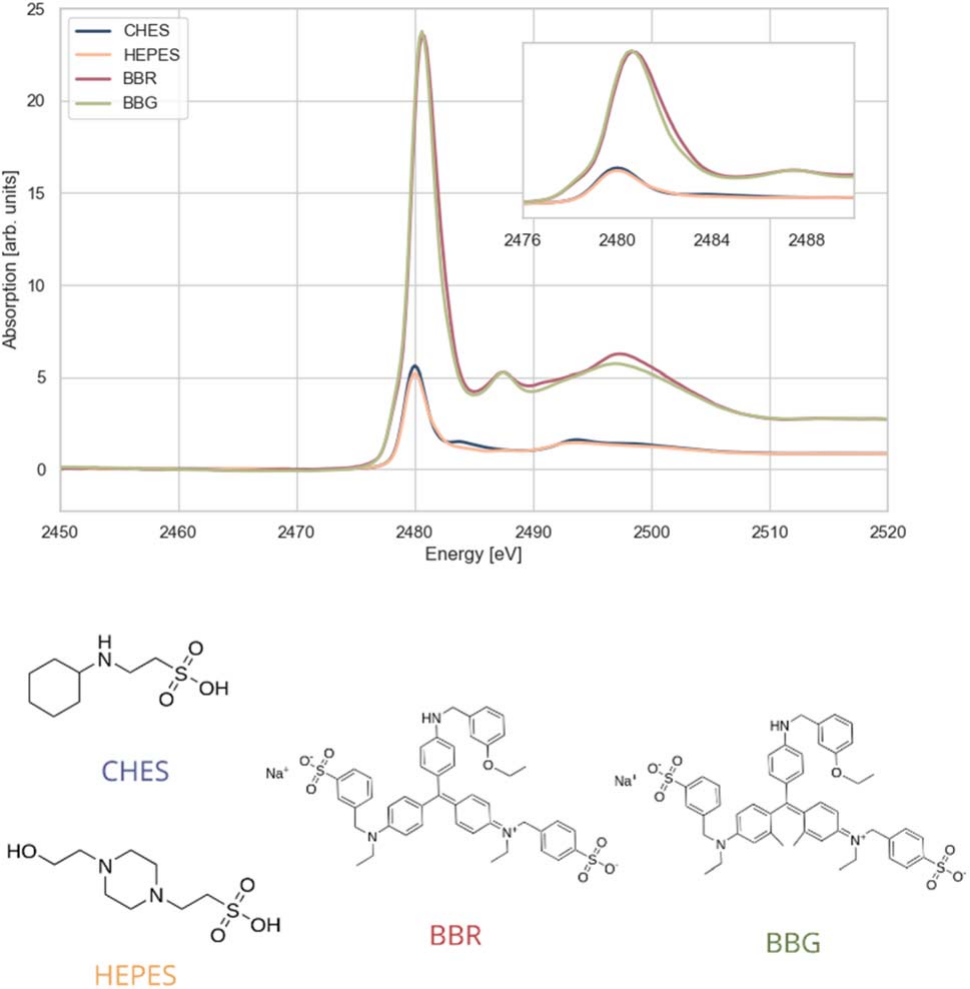
Sulphur K-edge XANES spectra of compounds with sulphur environment SO_4_^2−^; SDS (sodium dodecyl sulphate), Li_2_SO_4_ (lithium sulphate), (NH_4_)_2_SO_4_ (ammonium sulphate), NiSO_4_ (nickel sulphate). The inset displays the white lines at a larger scale.

The final category of compounds includes representatives of the –SO_4_–R group. This group comprises three salts—ammonium sulphate, lithium sulphate, and nickel sulphate—as well as SDS. The white line position for these compounds falls within the range of 2481.3–2482.0 eV (Fig. 5). The shapes of the features centred around 2497.0 eV in the spectra of the samples with cations in oxidation state +1 are similar to each other and clearly differ from the corresponding features in the spectrum of NiSO_4_, with Ni in oxidation state +2.

In SDS, sulphur is part of a sulphate ester group (–SO_4_^−^) bonded to an alkyl group through the oxygen atom, which breaks the symmetry of sulphur bonding environment comparing to the free sulphate ion. This breakage results in the orbital degeneracy which is reflected in the observed splitting of the white line and slight shift towards higher energy.

Nickel sulphate is unique among those salts as its sulfonate group is coordinated to a divalent nickel ion, resulting in the lowest white line maximum position (2481.3 eV). This can be attributed to nickel's lower electronegativity compared to the lithium and ammonium ions. In contrast, the lithium ion, with its relatively high electronegativity, shifts the white line maximum closer to that in the SDS spectrum (2481.9 eV).

**Fig. 6:**
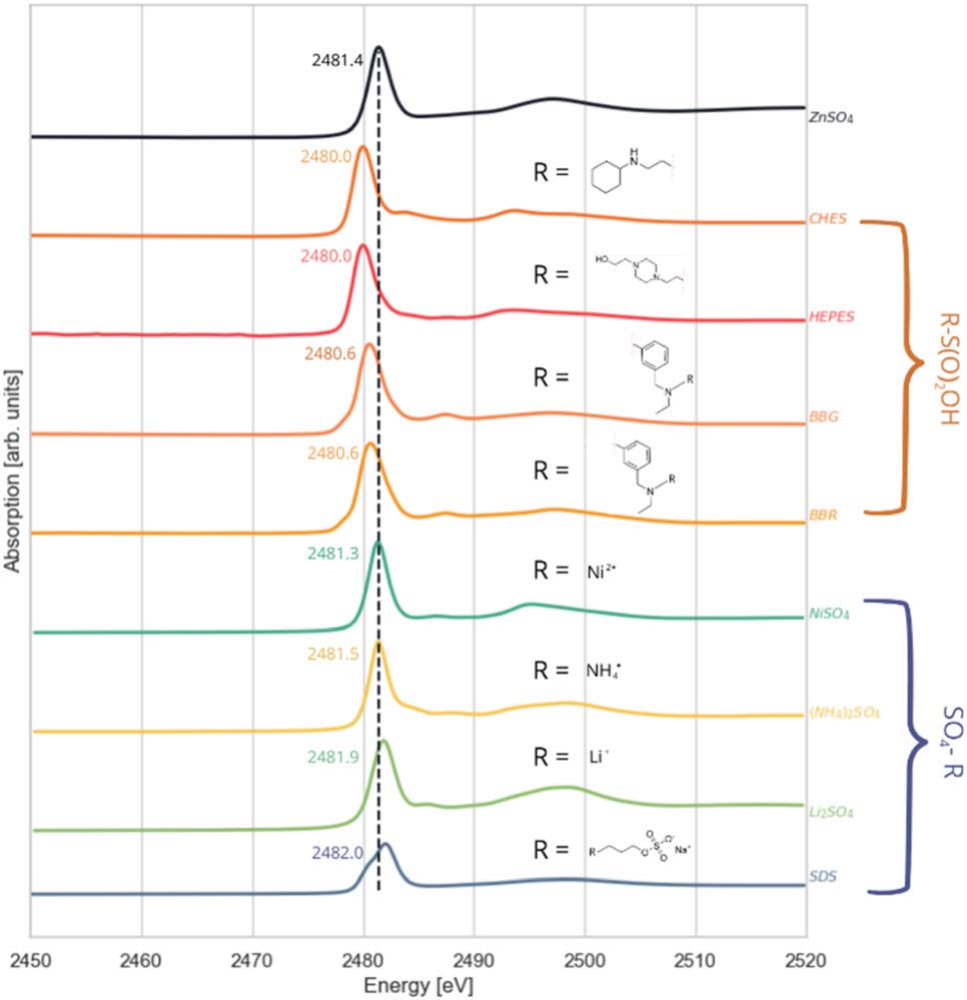
Sulphur K-edge XANES spectra of compounds with sulphur environment R–S(O)_2_OH and SO_4_–R.

The spectra for the different compounds are shown in Fig. 6. These data illustrate that the sulphur environment in the R–S(O)_2_OH group induces a shift towards lower energies compared to the reference compound ZnSO_4_.

**Fig. 7:**
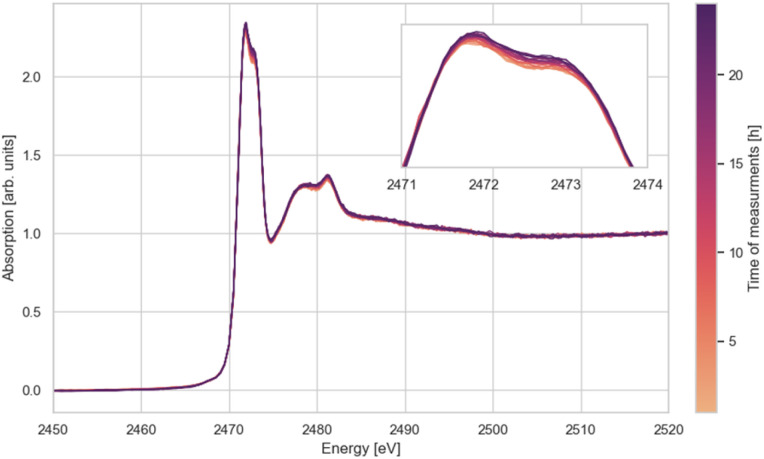
Impact of the number of measurements on sulphur K-edge XANES spectra of lysozyme.

In general, when measuring XAS spectra of biological samples one should check the results for possible radiation damage. As an example, Fig. 7 shows XANES spectra of the protein lysozyme, measured over 25 single scans in aqueous solution under stationary conditions.

The results in Fig. 7 show that even in the case of this protein, known to be highly sensitive to radiation damage by X-rays, no measurable influence of irradiation on the XANES spectra was observed over a long time. This liquid phase substance was measured in a closed sample cell without interacting with the environment in the sample chamber. In general, compared to many other beamlines, the probability for radiation damage to occur at ASTRA is fairly low, since the light source is a bending magnet (low overall flux compared to insertion devices), and the beam is unfocused, *i.e.* the intensity is distributed over a relatively large sample area (beam profile: 1 mm high, 10 mm wide).

**Fig. 8:**
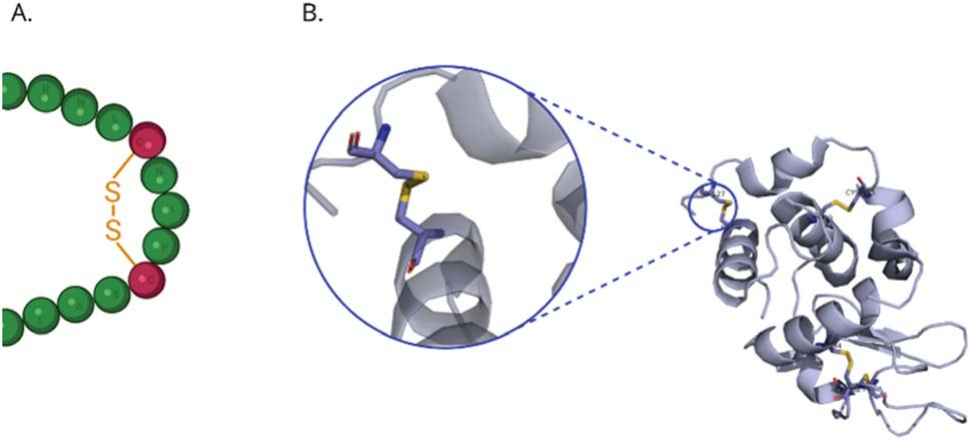
Disulphide bridges in the structure of lysozyme (A) schematic representation of the amino acid sequence, (B) structure of lysozyme with disulphide bridge marked.

In the lysozyme molecule, the sulphur atoms are linked to 8 cysteine residues forming four disulfide bridges, which define the structure of the protein and are essential for maintaining its 3-D shape (see Fig. 8). The enzyme contains four disulphide bridges. If the protein undergoes denaturation, for example due to radiation damage, a significant change in the spectrum would be evident because of the disruption of the 3-D structure and the breakdown of the disulphide bonds.

### Example of the use of a library of reference spectra

#### Linear combination analysis

Further studies, involving investigation of more complex biological systems such as proteins, require an extensive library of compounds representing different atomic environments of sulphur in the investigated samples. Biological complexes, especially in liquid environment, are often dynamic and may be hard to interpret without appropriate references. As an example of the use of the library compiled for this project, X-ray absorption spectra of mixtures were analysed using linear combination fitting (LCF). This method is mainly used for XANES data analysis based on the additive nature of the X-ray absorption of each sulphur species in the sample. Software packages like *e.g.* Athena (used for this project) determine the contribution of each reference spectrum to the spectrum of the sample based on a least square fitting algorithm.^50^

**Fig. 9:**
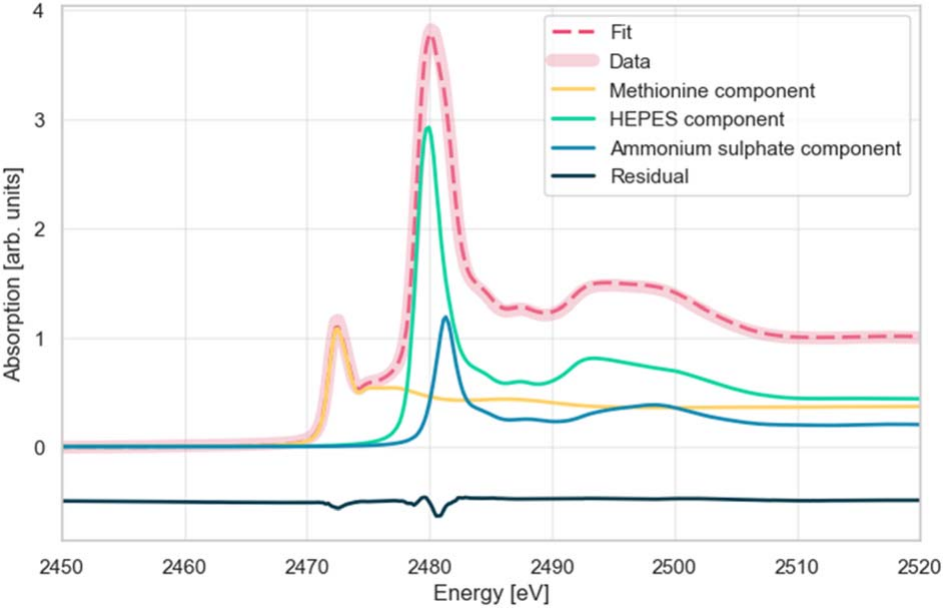
Sulphur K-edge XANES spectra of reference compounds compared to the spectrum of a mixture of these substances (red) and the result of linear combination fitting (dotted red).

For this project, reference spectra of compounds with sulphur in different chemical environments were measured. The collected spectra (Fig. 9) show how the position of the white line peak changes depending on the sulphur oxidation state. Furthermore, a mixture of the reference compounds was prepared to verify that the absorption spectrum of this mixture and the linear combination of reference spectra, determined by the data analysis software, coincide (Fig. 9). From the LCF procedure the following relative concentrations in the mixture were obtained: methionine 36,7%; ammonium sulphate 19,6%; HEPES 43,6%.

Fig. 9 shows the XANES spectrum of the mixture, the spectra of the pure components multiplied by the respective weights resulting from the fit and their sum (fit result). The residual, *i.e.* the difference between the spectrum of the mixture and the fit, is also shown. The maximum absolute value of the residual is approximately 3.6%. The root mean square of these differences for each point is 0.00114. The above parameters, together with the reduced chi-squared statistic value of 0.00078, show that it is possible to obtain very good fit results for the data recorded at the ASTRA beamline using the linear combination method.

#### *In silico* calculations

For simulations, a FDMNES program for calculating theoretical XANES spectra from crystallographic data based on the finite difference method was used.

Sulphur K-edge XANES spectra are dominated by dipole-allowed electronic transitions, where the angular quantum number changes by one (Δ*l* = ±1). When absorbing X-rays, the 1s core electron is excited to the lowest unoccupied molecular orbitals (LUMO). The type of LUMO orbital depends on the chemical environment: sulphur has the electronic configuration [Ne]3s^2^3p^4^ in S(0) compounds, [Ne]3s^2^3p^6^ in sulphides S(2−), [Ne]3s^2^ in sulphites S(+IV) and [Ne] in sulphates S(+6). Therefore, the absorption spectra are strongly affected by shielding of the nucleus charge by the electrons. The more electrons are ‘withdrawn’ from the sulphur atom by a bonding partner with high electronegativity, the lower the shielding and the higher the energy required to excite 1s electrons, resulting in white line shifts to higher energy.

**Fig. 10:**
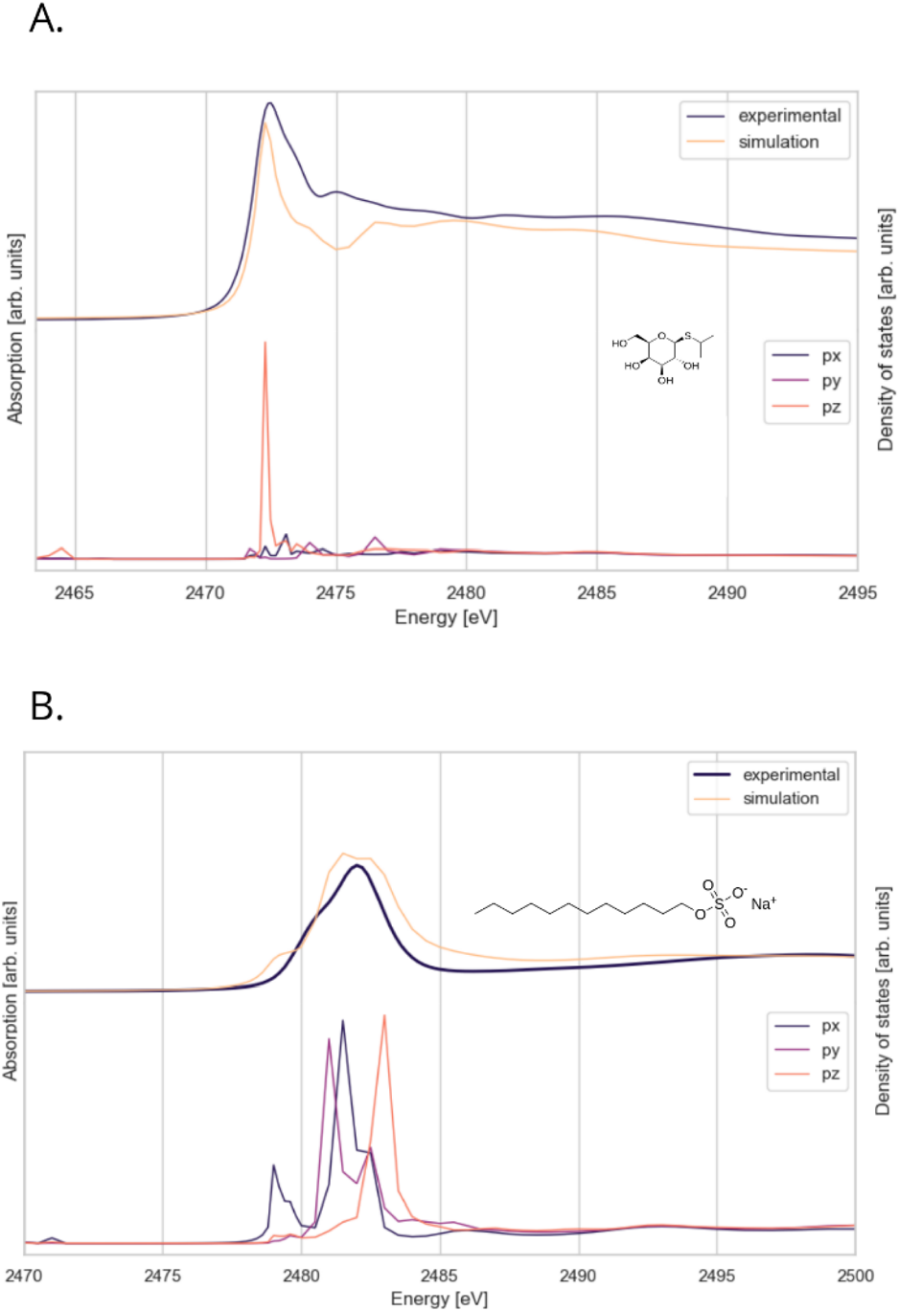
(A) Experimental absorption data and spectra of IPTG simulated with FDMNES (left axis), correlated with the density of states (right axis), showing the occupancy of different molecular orbitals present in the molecule. (B) Experimental and simulated spectra of SDS, and electronic transitions in the orbitals responsible for the features in the spectrum.

For in-depth analysis of the experimental data, DFT molecular simulations were used. Fig. 10 shows experimental and calculated XANES spectra of the selected sulphur compounds. In the case of IPTG the spectrum exhibits a very sharp white line peak without pre-edge features. Formally, the 3p shell of sulphur in IPTG should be fully occupied. In case of transition metal sulphides, the 1s → 3p transition is usually explained by hybridization of some sulphur 3p with metal 3d orbitals creating unoccupied 3p-type states.^57^ The features in the IPTG spectrum are most likely due to similar effects, specifically sp^3^ hybridization with carbon atoms. According to the molecular simulations, the white line is almost entirely due to p_*z*_-hybridized molecular orbitals, while in the post-edge energy range the contribution of all p-type orbitals is similar. On the other hand, in SDS sulphur has a different environment, giving rise to a much broader white line. Calculations show a small pre-edge feature at 2479.0 eV, which is absent in the experimental spectra. Unlike for IPTG, the contributions of all p-type molecular orbitals are similar. The main white line in the S(VI+) and S(IV+) XANES spectra is attributed to the 1s → 3p transition (σ* type).^58^ For SO_4_^2−^ and SO_3_^2−^ this feature is reported to result from electronic transitions into various molecular orbitals mainly formed by O 2p and S 3p atomic orbitals, and to a lesser extent by S 3d.^58^ Post-edge features in sulphur K-edge XAS spectra are generally attributed to multiple scattering resonances.

These two examples demonstrate that, in addition to ‘fingerprinting’ and linear combination fitting, molecular modelling can be a powerful tool for a detailed analysis of sulphur K-edge XAS spectra.

## Conclusions

The aim of this study was to demonstrate the necessity of creating a library of X-ray absorption spectra of sulphur-containing reference compounds for further biological research. We compiled an XAS spectral library of various sulphur-containing chemical reagents and metal salts. By creating the library and specifying the spectral ‘fingerprints’ of individual reagents, it will be possible to investigate the interactions of biological compounds (for example, proteins) with ions, assess the pollution of biological samples, and determine contaminants, especially as contamination can impact the structure of biological substances. This is crucial, since in many biological systems it is not possible to eliminate the presence of sulphur, as it is abundant in amino acids, cofactors, buffers, and ligands.

It is known that buffers can affect the structure of the biological reagents studied, an example being crystal formation. Some proteins crystallize only in certain buffers, since they remain most stable therein.^59^ With XANES studies, the relationship between proteins and buffers can be investigated.

Due to the rapid pace of measurements, simplicity of sample preparation, and clarity, XANES spectroscopy is an efficient method for testing biological compounds and can be used to assess whether a specific chemical reaction has occurred, in what manner, and to what extent.

Sulphur was chosen for this XANES study as it occurs in various chemical states, and the energy positions of the corresponding white lines are spread over a broad energy range and can therefore easily be distinguished with the energy resolution typically available at XAS beamlines. Accordingly, XANES spectroscopy allows an accurate analysis of the sulphur speciation in the investigated compounds.

As one of the first user groups recording XAS spectra in the tender energy range at the ASTRA beamline we had a chance to test the beamline's capabilities in the context of biological research. ASTRA is a versatile experimental station allowing experiments in a broad energy range, covering the X-ray absorption edges of numerous chemical elements, and different sample environments. In our opinion, the beamline is well prepared for biological research and poses no risk of radiation damage, even for sensitive protein samples. It is worth to emphasize that the possibility to measure XANES spectra at the K-absorption edge of sulphur and several even lighter biologically highly relevant chemical elements (down to Mg) make the ASTRA beamline a powerful tool in life sciences studies.

The project will be further developed to use the ASTRA beamline as a preliminary stage of the planned studies of metal-dependent enzymes. At the outset of this project, when the ASTRA beamline was in its early stages of operation, its measurement capabilities were limited to solid samples. However, as the beamline has developed, it has become capable of measuring liquid samples. Future studies will focus on utilizing this expanded capability for the measurement of liquid specimens and the work on the library of sulphur-containing compounds will be continued and all collected spectra will be publicly available. In the future, we plan to extend the library with spectra of compounds containing further chemical elements of interest for biological research.

## Data availability

Data from the XANES library is available at the ASTRA beamline and can be requested from the corresponding author or using DOI: http://doi.org/10.57903/UJ/VRJ85T. The code for XANES library and spectra analysis is available at the ASTRA beamline and can be requested from the corresponding author

## Author contributions

AK: data curation, investigation, formal analysis, writing original draft, visualization, resources, library website creation. JS: project administration, writing – review & editing. GG: methodology, data curation, visualization, writing – review & editing. AM: conceptualization, supervision, writing – review & editing, formal analysis. AS: data curation, formal analysis, visualization. MP: software. HL: writing – review & editing. MK: supervision.

## Conflicts of interest

There are no conflicts to declare.
